# Healthcare professionals’ and patients’ perspectives on consent to clinical genetic testing: moving towards a more relational approach

**DOI:** 10.1186/s12910-017-0207-8

**Published:** 2017-08-08

**Authors:** Gabrielle Natalie Samuel, Sandi Dheensa, Bobbie Farsides, Angela Fenwick, Anneke Lucassen

**Affiliations:** 1 Brighton and Sussex Medical School, Falmer, BN1 9PX UK; 2 Department of Educational Research, Lancaster University, Lancaster, UK; 3 Clinical Ethics and Law, University of Southampton, Southampton General Hospital, South Academic Block, Tremona Road, Southampton, SO16 6YD UK; 4 Wessex Clinical Genetics Service, University Hospitals Southampton Trust, Southampton, UK

**Keywords:** Consent, Autonomy, Genetic testing, Genomics, Virtue ethics, Patient decision-making, Ethics

## Abstract

**Background:**

This paper proposes a refocusing of consent for clinical genetic testing, moving away from an emphasis on autonomy and information provision, towards an emphasis on the virtues of healthcare professionals seeking consent, and the relationships they construct with their patients.

**Methods:**

We draw on focus groups with UK healthcare professionals working in the field of clinical genetics, as well as in-depth interviews with patients who have sought genetic testing in the UK’s National Health Service (data collected 2013–2015). We explore two aspects of consent: first, how healthcare professionals consider the act of ‘consenting’ patients; and second how these professional accounts, along with the accounts of patients, deepen our understanding of the consent process.

**Results:**

Our findings suggest that while healthcare professionals working in genetic medicine put much effort into ensuring patients’ understanding about their impending genetic test, they acknowledge, and we show, that patients can still leave genetic consultations relatively uninformed. Moreover, we show how placing emphasis on the informational aspect of genetic testing is not always reflective of, or valuable to, patients’ decision-making. Rather, decision-making is socially contextualised – also based on factors outside of information provision.

**Conclusions:**

A more collaborative on-going consent process, grounded in virtue ethics and values of honesty, openness and trustworthiness, is proposed.

## Background

Consent has been argued by some to be the foundation of contemporary medical ethics, and a pinnacle of ethical clinical practice. Underlying consent is the notion that patients can make autonomous decisions and that in doing so they are protected, or can protect themselves, from harm. For consent to be valid, adequate information must be provided to a patient about any proposed course of clinical action, including its alternatives, benefits and risks.

In some areas of medicine the relationship between information provision and maintaining patient autonomy are (more) clearly defined, being related to the goals of care for a particular patient at a particular time. For instance, for a surgical procedure that has a clear beginning and end, patients can be informed of the benefits, risks and alternatives, allowing them to make an informed autonomous choice. In other areas of medicine, however, the action for which consent is sought is less sharp, and disputes remain among academics and healthcare professionals (HCPs) about what, and how much information is required to achieve adequate consent and ensure patient autonomy, especially when the goals of care may be blurred [1]. Clinical genetic or genomic testing provides a good example – particularly broad and untargeted tests, such as comparative genome hybridisation (‘arrays’), and whole-exome and whole-genome sequencing. Information about the benefits, risks and possible outcomes of this testing may be uncertain and/or only accrue over time. Indeed, the Joint Committee on Medical Genetics (JCMG) guidance is acutely aware of these issues, noting within its guidance that being fully informed is not possible in this setting [2].

In this paper we argue in line with this guidance that in clinical genetic testing, the desire of HCPs to maintain patient autonomy and prevent harms has involved too much emphasis on providing information to patients - the ‘informational’ aspect of consent. We challenge the idea that in order to make an autonomous and informed decision about clinical genetic testing, patients need to know all the specific information about the test. An information-loaded consent framework is neither possible nor useful in meeting the aim of enhancing autonomous decision-making in this setting. Rather, without appearing paternalistic, and without thinking they are harming patients, HCPs must realise, and convey to patients, that uncertainty exists in this area of medicine. Not having, or giving, all the specific information about genetic testing outcomes does not mean patients are ‘uninformed’.

We also go further and argue that consent should be viewed as relational, and as an on-going collaborative decision-making process between the HCP and patient. This process should be based on trustworthiness, openness and honesty, and as such can be seen as rooted in virtue ethics. The extent to which these virtues are embedded in clinical decision-making will thus go some way to tell us about the ethical nature of the process.

To build our argument, we draw on a set of focus groups with HCPs working in genetic medicine and a set of interviews with patients who have sought genetic testing. We guide the reader through our empirical findings.

### Consent in genetic and genomic medicine

To set the scene, we summarise the existing discussions about consent in clinical genetics highlighting four specific issues.

First, the predictive nature of genetic information raises issues about what to test for, when, and whom to test, for example, the question of whether to test children, or analyse their already-sequenced genome, for indications of currently far-off, adult-onset risks. Second, the innovativeness, and growth, of more detailed genetic analysis raises questions about the increasing chances of finding genetic predictions or diagnoses that are not related to the reason for the test (so-called incidental findings, or IFs): for example, risks for hereditary cancers amenable to risk-reducing interventions. Specifically, questions arise about how these might be anticipated and incorporated into the consent process and even whether they should be reported if not specifically sought [3–9]. Third, the familial nature of (some) genetic information raises issues about whether confidentiality can best be viewed at the individual or familial level and whose responsibility (if anyone’s) it is to communicate risk to at-risk relatives [10–12]. Finally, genetic testing’s often traversing role across research and clinical practice [13] raises issues about the ‘therapeutic misconception’, and whether patients expect to receive clinical information from their participation in a research study, or expect that their clinical tests will be further researched.

Because of the complex issues and implications surrounding genetic testing, genetic HCPs often provide genetic counselling in the way of education, guidance, and pre-and post-test information about the risks, benefits, limitations, and implications of tests (including the potential for IFs), as well as data storage and data usage (e.g., use in quality control or research) [5, 14]. This approach ostensibly facilitates patient consent to genetic testing and is viewed as a positive ethical feature. Indeed, evidence suggests genetic counselling improves knowledge and decreases anxiety, distress and depression [15]. Even so, concern remains about the lack of feasibility, applicability, or benefit to patients of receiving *all* of this information during the consent process. This is true of genetic testing in general, but especially relevant to broader genome analysis [16, 17]. Some HCPs, for example, understand that patients cannot always be expected to fully understand the range of different possible results and implications of testing because the analysis undertaken can often be open-ended and results uncertain. Others have gone further, and argued that too much detailed information can overload patient understanding [18, 19] and undermine autonomous choice [20]. Depression, anxiety, or desperation may lead to incomplete understanding of the information given [19].

### Relational ethics

An emerging critique from the social sciences and anthropology is a questioning of the informational aspect of consent [13, 21–29]. Central to these critiques is a problematisation of the assumption that patients are autonomous agents who make rational choices based on neutral information. There is recognition that autonomy is relational, and factors beyond information must also be emphasised as relevant to decision-making. These factors relate to an individual’s social, cultural, emotional and/or personal or familial context [22] and include, for example, patients’ expectations of the clinical encounter, the nature and severity of the illness, and clinician-patient interactions and relationships [24, 30, 31]. Viewing consent in a way that does not consider the relational aspects of autonomy leads to an ‘*empty ethics’* [24] and strips the principle of consent away from its social context. Trust and hope, for example, are perceived as important factors for decision-making, where trust is seen to be placed in clinicians, who are viewed as protecting patients against harm [18, 32]**.** Interestingly, similar findings have also been noted in the research/bio-banking setting, where trust and hope are entangled in the belief that research will produce (sometimes personal) therapies in the case of clinical trials [33], and offer societal benefits by advancing medicine [34–36]. Regarding practices that combine clinical practice and research, a survey by Genetic Alliance UK showed that while 38% of respondents trusted private companies to do research using their health data, 80% trusted the UK’s National Health Service (NHS) to do so [37]. In terms of the research aspect, the perceived relationship between research and participant has been shown to play an important role in shaping preferences regarding consent [38].

In this paper, we draw on some of the arguments outlined so far to propose that it is better to move away from an approach to consent that places autonomy, and the need for information, as the central reason for consent in genetic testing. Rather, these should be seen as equal among other principles to be upheld within a more relational approach to consent: those of trustworthiness, openness and honesty. While HCPs in genetic medicine are, as we will show, to some extent already adopting such relational frameworks of consent, many are not and such a framework needs to be acknowledged more extensively and at a more regulatory level to ensure that all HCPs conducting clinical genetic tests are aware of best practices.

Our arguments are particularly relevant and timely for three overlapping reasons. First, HCPs who do not specialise in genetic medicine, and who may have little experience with seeking consent for clinical genetic tests, are being increasingly encouraged by NHS to adopt such testing into routine clinical practice. We consider it important that they take a relational, rather than a solely informational, approach. Second, although unclear whether and how it might affect clinical genetics/genomics, a recent UK legal ruling (*Montgomery* vs *Lanarkshire*), which states that a doctor must take ‘*reasonable care to ensure that the patient is aware of any material risks involved in any recommended treatment, and of any reasonable alternative or variant treatments*, might mean that now more than ever, clinicians consider more information to be better – indeed that without a barrage of information about possible outcomes from genomic testing, consent might not be valid. It is important to highlight the shortcomings of such legal rulings in the practice of clinical genetics/genomics, particularly because of the possible current and future uncertain predictions it might make. Third, projects are launching worldwide that combine research and clinical care and aim to integrate whole genome sequencing into clinical practice. The UK 100,000 genomes project for example takes an informational approach to consent, whereby patients are given a 40 min-two hour appointment, an eight-page information leaflet, and a five page consent form to sign multiple times. Such an informational approach to consent runs the risks of turning future clinical practice into a disclaimer interaction that does little to enhance the validity of consent about unexpected, uncertain or future predictions.

## Methods

### Methodological rationale

This paper draws upon patient interviews and HCP focus groups conducted by the second author, SD, as part of a larger project about consent and confidentiality in genetic medicine [32]. For this paper, the first author, GS, analysed the interview and focus group data.

GS was initially unaffiliated with the larger project. However, with the aim of forging a new collaboration between SD, AL and AF, GS was given access to conduct a secondary analysis of SD’s data which related, but was not directly relevant to, the larger project’s research questions. Concerns relating to the secondary use of qualitative data have been documented and include issues associated with contextualisation and data interpretation [39]. Given these, GS was cautious proceeding along this path and, indeed, during her analysis, she experienced many of these concerns. As a result, she became more affiliated with the original research team, drawing on their knowledge, experience and interpretation of the data to ensure the findings reflected the data meaningfully. All data interpretation was thus in collaboration with SD, AL, and AF to ensure that the emerging themes represented their experiences, and were reflective of their views of the data.

### Recruitment and sampling

#### Patients

In 2013, information about the research project was sent to collaborators in three large UK genetics centres. These centres posted the information onwards to all recent patients seen for genetic testing. Information was also posted on online support groups for hereditary cancers and cardiac conditions. These conditions were chosen as they are the most commonly seen in genetics services; there are available risk-reducing interventions; and because they have an inheritance pattern that means family members could be at risk—an important consideration because the original project explored confidentiality and family communication. SD conducted 33 semi-structured interviews with adult participants. Interviews lasted around one hour. The interview schedule has been reported previously, and comprised general, open-ended, and non-leading questions designed around the research questions and empirical and conceptual literature [32]. Some of these interviews were not used for re-analysis: in two the recording failed and SD’s notes were unsuitable for reanalysis and several participants had not consented to their data being used in future research.

#### HCPs

UK HCPs involved in genetic testing were invited to take part in the research (2013–2015). Recruitment was purposive, proceeding via presentations at professional meetings, and emails to heads of departments for dissemination to colleagues. Eighty HCPs agreed to participate (representing *n* = 14/24 regional UK genetic services), and 16 focus groups were held. HCPs included genetic counsellors (*n* = 37); clinical scientists (*n* = 16); consultants in clinical genetics (*n* = 8); clinical genetics registrars (trainees) (*n* = 8); nurses working in a genetics team (*n* = 4); fetal medicine professionals (*n* = 4); family history coordinators (*n* = 2); and a nephrologist (*n* = 1). Where possible, focus groups consisted of real-life teams to provide an understanding of the context in which HCPs work and make decisions. Discussions were facilitated by SD, audio-recorded, and lasted approximately one hour. A detailed account of the methodology has been reported previously [32].

#### Data analysis for this study

Transcripts were analysed using aspects of grounded theory methodology. Analysis had two main iterative stages: (1) description of each transcript, which formed the basis of the forthcoming abstraction and analysis, and (2) coding and creating themes. Following (1), two focus groups with fetal medicine professionals and two with research scientists were excluded from analysis as these professionals did not seek consent for genetic tests. One pilot focus group was also excluded as it had little relevant data. Twenty-one patients were excluded from analysis for various reasons including those mentioned above: some participants had not had a genetic test or had one many years previous; and one had tested for Huntingdon diseases so did not fit with the profile of the other participants. Some transcripts contained insufficient or no relevant data. In the end, 11 HCP focus groups and 12 patient interviews were retained for analysis. Data were managed in NVIVO.

Analysis was initially microscopic, in that in involved a line-by-line analysis, with a particular focus on participants’ discussions of consent and decision-making for genetic testing. Constant comparisons were made between transcripts, between patients and HCPs, and between findings at each stage to those at subsequent stages. These comparisons facilitated coding, of which there were three iterative aspects: open coding, axial coding, and selective coding. Open coding involved labelling meaningful aspects of text, including concepts (the building blocks of theory and argument) and processes (the evolving and dynamic actions and interactions between participants, other people, and their environments, over time). Axial coding involved categorising open codes—grouping similar codes and interrogating the way they related to each other, which helped us to form the arguments underpinning the themes. During this process, we revisited the transcripts to ensure our emerging arguments reflected the data. Selective coding involved the integration and refinement of these arguments [40].

## Results

Findings are divided into two sections. First, we draw on focus group data to highlight HCPs’ efforts to adequately inform patients to allow them to make decisions about genetic testing. Second, we use focus group and interview accounts to highlight how, despite this, patients do not always understand the specifics of the information provided and, moreover, understanding this information is not always reflective of, or valuable to, patients’ decision-making. The final discussion section draws these sections together.

### Focus groups with HCPs: How genetic HCPs consider the act of consenting patients

In this first section we show how HCPs viewed consent as both being integrated in patient discussions in clinic appointments and as being the signing of a consent form. While HCPs placed some value on the signing of consent forms, ensuring patient understanding of the information provided prior to testing was considered of paramount importance.

#### HCP views on the importance of information-provision for consent

HCPs understood that patients often arrived at a consultation with little understanding about testing, and spent much of the consultation explaining the implications and exploring patients’ views and feelings about having the test. The consent process was, in this way, an integral part of the consultation: ‘*its giving them the information in a way they can understand it enough to make a decision that’s appropriate for them’* (FG3P1); ‘*we spend 45 minutes essentially consenting a patient for a test...a lot of the time that’s the purpose around the consultation*’ (FG6P3).

This perceived importance by HCPs to adequately inform patients stemmed from a belief that genetic, and especially genomic, testing was more ethically troublesome than clinical investigations in other medical specialities: *‘genetic tests are different [to other tests] because they give permanent information about you [and] indications for your relatives, so it’s harder to see them in isolation, and I think sometimes, not only the patients, but doctors forget that’* (FG15P5). Indeed, maybe because of this, genetic HCPs thought other HCPs might pay less attention to such issues:

- FG7P2: there are some people that have taken a lower, not a lower view, but a less stringent view of consent, and possibly don’t think about it as much as we do….
- FG7P1: I think a GP for example would do perhaps a battery of tests and wouldn’t think twice probably of saying, ‘well actually we did an anaemia test but your blood sugars are up’, whereas we [genetics] would be [more] worried about finding something else.

Corroborating their strong desire to ensure patients were informed were HCPs concerns that the ever-increasing mainstream specialities now ordering genetic tests might not grasp the ethical implications of such test results, and as a consequence, patients seen by these HCPs might not understand genetic testing or its consequences: *‘that’s our greatest anxiety, because genetic testing in the very near future here is going to come online to other specialists without any genetics support..[..]..where the whole issue of genetic testing and consent [how best to reveal] results just doesn’t ever sort of reach consciousness’* (FG13P1). Previous research has also shown genetic HCPs to have such concerns [5].

Therefore, rather than viewing consent as ‘*nothing more than a set of procedures to be followed*’ [18, 41], HCPs’ emphases were heavily weighted on ensuring patient understanding of genetic information. They perceived their ‘ethical awareness’ to be related to the special nature of genetic information, which stands apart from other forms of medical data in terms of its permanency and familial nature.

#### HCP views about patients signing a consent form prior to testing

Given the amount of information they had to convey, HCPs saw consent forms as sometimes helpful because they prompted and structured their discussions with patients. For many HCPs, forms were useful for documenting consent and creating a necessary summary of these discussions, in which they had explained the concept of genetic testing and its implications for the patient and family members. The form acted as a reference to what patients were consenting to and why: ‘*it also gives the patients a kind of anchoring point as well. That they feel 'alright, this is where I’m going with thi*s'’ (FG6P1). A signed consent form was also thought to provide some reassurance that HCPs were more protected against any future potential professional or legal ramifications, although HCPs themselves cast doubt on this assertion: *‘in a way it’s like a legal document but then it’s not legally verified; it’s for our peace of mind essentially’* (FG101P1). One participant expressed feeling ‘*a lot happier if I’ve got that person’s signature*’ (FG16P3). These findings are in keeping with previous research highlighting patients’ beliefs that the primary function of consent forms is to protect hospitals [42].

The consent form also served a more ‘*practical*’ (FG6P2) purpose by providing documentation about potential contact of other family members if relevant, perhaps by other HCPs (*‘it’s nice to have it documented, because when we’re not here and someone else looks at the file’* (FG8: specific participant inaudible). Such a situation could also arise in the future, years after the patient had consented to genetic testing: ‘*if…there’s a sample that was tested fifteen years ago, and no-one has documented any consent about whether or not that information can be shared…just looking at things in the long-term, I do think it can be really important information’* (FG6P1).

A handful of participants placed little value on the form or the need to complete it before testing. For them, documenting the decision-making process between HCP and patient was important, but could be recorded just as well in clinical notes: ‘*we’ve got a consent form, which we don’t always use to be honest, but we’ll still document’* (FG7P2). Some HCPs perceived the discussions surrounding consent, rather than any written documentation, as paramount to ensuring patients were informed about testing: ‘*I think it is good practice to take written consent, but…it’s never a substitute … for actually making sure patients understand’* (FG6P3). Such differences possibly reflected the use of genetic testing for diagnostic purposes—during which clinicians may be less concerned with distinguishing genetic testing from any other clinical investigation for which written consent would not normally be sought—as opposed to predictive genetic testing, where documenting consent is considered more ethically appropriate because of the novelty, complexity and uncertainty of many such predictions.

### Problems with placing ethical emphasis on the informed aspect of consent

In this section we question the ethical weight placed on the informed aspect of consent by looking at three issues. First, despite HCPs’ efforts to ensure patients understand the process of genetic testing and its implications, they observed that patients often left consultations with limited comprehension. Second, alongside clinical information, emotional, social and situational issues also played a prominent, often intertwined, role in patients’ decision-making. Third, patients discussed, and HCPs observed, that clinical information given during the consultation can be of limited importance or value to them.

#### Patients do not always fully understand or retain information about genetic testing

Despite the attention they paid to ensuring patients were informed, HCPs expressed concern that sometimes families were still unable to understand information. FG6P3 explained how ‘*the majority of patients don’t know they’ve had a genetic test, even though they’ve signed a consent form’*. Others discussed how patients, even if they had initially understood information, were later unable to remember it, which made them doubt the patient’s level of understanding. Indeed, FG5P3 remarked that it would be incorrect to assume the information relayed and explained to patients had been duly considered: *‘you think you've explained it…they nod at you nicely...we can't assume that because it's easy for us it's easy for them. It takes ages...it's just the penny has not dropped …’*. One HCP talked about the consent form as evidence to remind the patient of their consultation (*‘a lot of them will say “I’ve never been to genetics”, and you know they have because…you’ve got information in the file’* (FG8P2)).

Interview and focus group accounts suggested a number of reasons to account for (potentially) poor understanding of genetic testing: the complexity of the information provided; the number of simultaneously-offered diagnostic tests making it difficult for patients to distinguish or process the difference between a genetic test and other non-genetic diagnostic procedures; and patients’ minds being too focused elsewhere during the consultation, for example on the emotional roller-coaster of their (or their child’s) life, to concentrate on genetic testing and its implications: *‘I do remember signing it; I don’t remember the talk before I did that. I was quite nervous’* (patient 6).

As such, although nearly all patients spoke about the importance of being informed (‘*information is power…by knowing things you can make decisions’* (patient 24)), and while some had spoken with clarity about the consent process (‘*they was [sic] quite good. They explained everything*’ (patient 17); ‘*she gave me a lot of information*’ (patient 10)), their comments corresponded to clinicians’ concerns: that difficulties in understanding meant that patients did not always leave the consent process fully comprehending the implications of their impending genetic test(s). For other patients, they might have initially understood the implications of the test, but could now not remember them – as patient 9 noted in relation to consenting to familial sharing of genetic data, *‘I don’t remember them saying anything particularly about that’.* This raises concerns about using a one-off information appointment as a gateway to consent for testing – something increasingly the case in NHS consultations for certain familial cancer predispositions, for example.

#### Consent and the need to consider emotional, social and situational issues

As touched upon above, in some instances a patient’s decision to have a genetic test was less based on the information provided to them, and less a case of them acting as rational autonomous individuals, weighing up information devoid of emotional and social context. Rather, their autonomy was relational: decisions were embedded in, and their rationality inextricably linked to, their emotional, cultural or social relationship with the world around them. The extract below highlights how HCPs thought for some patients there was ‘so much going on’ for their families at the time of diagnosis, it was difficult for them to adjust and consider what they were consenting to:

- FG14P4: …at the time of diagnosis there’s so much going on…
- FG14P3: Yeah, and then once they’re adjusting they start taking it on board. And we simply, we don’t necessarily see them at that point do we?
- FG14P4: No, no, that seems to be what we’re sort of identifying here isn’t it, it’s that in the shock or the trauma of the initial situation people understandably, are going to be processing information.
- FG14P3: And just thinking…what’s wrong with my child; that’s what their focus is.

Patients corroborated that it was difficult to emotionally process information at the consultation. One noted that deciding to have the test on a re-visit rather than an initial visit to the HCP allowed for such emotional processing to take place: ‘*I think it was probably right that there was a gap between the two [consultations], to give you time to process it all [before deciding to have the test]’* (patient 18). Others noted that because emotional processing often did not occur until after testing, the implications of a genetic test were not always thought through even at the time of consent. Patient 25, for example, said she had not considered the implications of genetic testing: the decision was clouded by one she felt more sure about—to have a risk-reducing mastectomy:

- Even though somebody [a HCP] might tell you [the implications of the genetic test], and I’m sure they did, you don’t, look past that test. You think “oh yeah I’ll have a test, so I really should know if I’ve got something”, but you don’t realise once the results [are there], then you have to make another decision that’s far more difficult.

These findings support previous research arguing that decision-making during consent is not just related to the provision of information (the ‘information paradigm’ [22]), but also its social context [5, 35].

#### Values important in patient decision-making

Many of the issues covered in the consent process, and those summarised on consent forms, were perceived by some HCPs not to ‘*actually matter that much to [the patients]’* (FG8P4). For instance, HCPs expressed that at least in some cases the anxiousness that surrounded giving consent for the familial sharing of information was related less to concerns about sharing genetic information, and more to social and personal concerns about sharing other, more personal, information from medical records.

- FG12P3: I’ve only come across one person who’s said you must destroy this sample…and that …to me it was kind of a more generalised anxiety rather than specific to the test that we were doing. I think [for] most people…it will be something like non-paternity or I had a termination for social reasons I don’t what anyone to know…don’t tell anybody. It’s more those kinds of things that people are most concerned about.
- FG12P5: Not about what’s happening to my DNA.

In addition, HCPs explained how patients seemed to have little concern about the future testing or use of their genetic material for ‘the benefit of others’: *‘I think most patients you talk to actually don't have a huge problem with their information being shared for the benefit of others and so on. I think it's a minority that has a problem that gives the public the view that everyone has a problem…*’ (FG3P2). Indeed, as has been highlighted by others [35, 43], the use of genetic information for purposes such as research was viewed positively (‘*I have no worries at all, and any information, any kind of research, it’s going to help future generations…and it’s so important’* (patient 12)), and at times, HCPs felt research was almost assumed by patients to be happening:

- FG12P5: I’ve had lots of people assuming we’re going to do research on their sample, “are you going to use it for research then are you?.....”
- FG12P8: Some people want us to do research don’t they?
- FG12P5: Yes
- FG12P8: And say well why aren’t you?
- FG12P5: Keep it, keep it, and do all the research!

Previous research confirms that views about the use of biological material for research purposes are often not related to, or based upon the provision of information during consent. Rather, they reflect a whole raft of relational and virtuous notions relating to altruism, solidarity, trust in medical institutions and clinicians, and a belief in the welfare state [22, 35].

Indeed, exploring these relational and virtuous notions a little deeper, HCPs and patients seemingly placed much value on the importance of openness and honesty in their relationship during the consultation (‘*you always tell your patient that's what you're going to do and you're always transparent about what you are doing and why you're doing it’* (FG5P1); ‘*I think it's openness. I think if…something hasn't gone quite ideally…I think if you're honest about it then they don't feel cheated*’ (FG3P1)). This valuing of openness sat alongside a perceived need for a trustful HCP-patient relationship (‘*I think it’s really important that your patient feels that they can trust the relationship that they’ve got with you’* (FG16P1)). Trust has been shown previously to be paramount in any consenting process [18, 38, 43], and here it was no different: as Patient 2 noted, patients needed to trust HCPs to behave in an ethically responsible way: *‘as long as that conversation is had…we have to trust the health professional to behave in a professional manner’*.

## Discussion

### Drawing the findings together

Our findings have shown that HCPs acknowledged, and patients expressed, the shortcomings of an informational focus on consent. That is, despite HCPs’ efforts to enhance patient autonomy and protect patients against harms by adequately informing them about the specifics of genetic testing, situations arise in which patients have little understanding or memory of the consent process. Decisions about genetic testing were made in social contexts enshrouded with emotions and other personal concerns, and in some circumstances, the information covered during consent was of little relevance or value to patients, especially if this information was not directly related to how the patient considered the goals of his/her care at any particular time. Our findings thus give credence to the notion that consent should be more than ‘*an information-based, intentional act’* [35](page 16), and that failure to embrace the social context within which decisions are made about genetic testing will lead to an ‘empty ethics’ [24].

We reiterate previous research that suggests that being informed about all the possible outcomes of clinical genetic testing is not attainable, and, moreover, not always reflective of the nature of consent or decision-making [27, 31]. We also argue that such fundamental issues, related to placing emphasis on the informed aspect of consent, cannot be solved – as some propose – by providing more time for consent [5] or by providing ever more levels of complex information or technological solutions. Indeed, HCPs noted that they often become ‘*tied up in knots’* (FG7P2; FG12P3) because of the complexity of options for receiving results (which results to receive; when; and how) and this chimes with contentions that more information is not always better [44]. And while we see merit in proposing various models for approaching broad consent to genetic testing, as has been done in the research arena (for example, offering patients options to choose between types of IFs using ‘tiers’ or ‘bins’ [17, 20],^1^ these models cannot solve the fundamental issues associated with the notion that consent is broader than the provision of information alone. This is because of the reliance of these models on the belief that providing information to patients allows rational autonomous individual decision-making.

Instead of viewing consent as the passing on of decision-making capacity onto a (rational and autonomous) patient by the provision of information, consent needs to be seen as an on-going collaborative relational process in which decision-making is shared between HCP and patient. While this collaborative relational approach has been suggested to be appropriate in the specific context of disclosing genetic test results to patient’s relatives [27, 31], it has received less attention in the context of decision-making for genetic testing. Such collaboration needs to emerge not only as a result of the HCP providing information to the patient, but of HCPs remembering that to patients, clinical information might be deprioritised in relation to other emotional, social and/or personal concerns, and therefore they may have less need or desire to understand it. In our study, HCPs recognised this need. In fact, there is a body of literature that argues moral stances, or ethical perspectives and decision-making, are not a priori. Rather, they are context specific and can only emerge once individuals are placed into particular social, emotional, cultural and/or personal contexts [35, 45].

This same literature states that in an institutional context the relationships formed in these situations can affect decision-making [35]. Extrapolating this idea would suggest that a collaborative relationship (a relational approach) between HCP and patient would provide a supportive and caring environment for the patients so they feel they can, with the help of their HCP, make the decisions that are best for them, given not only the stage they are at in terms of diagnosis, but also their personal, social, emotional and cultural contexts. Without such a relationship, there is the danger that patients may make decisions during consent that do not best reflect their circumstances or wishes.

Furthermore, our findings suggest that this collaborative effort should be dependent on certain HCP characteristics. We note three here – trustworthiness, openness and honesty – though note that others could be reasonably drawn from the findings.^2^ HCPs viewed themselves as needing to embrace such characteristics to ensure the process-led approach to patient decision-making remained respective of patient’s relational (emotional, cultural, social) situations. Table 1 contains definitions of these virtures, an example of how they might help in a genetic tesing context, and illustrative quotes from our HCP participants. These findings resonate with notions of virtue ethics, ie., that there are certain virtues that HCPs need to display to build a relationship with their patients and ensure they are considerate of the consent process. Put another way, by drawing on the virtues of, for example, trustworthiness, openness and honesty identified in our analysis, HCPs can build a relationship with patients that extends beyond information provision, to one in which there is an understanding of relational autonomy. HCPs can then engage with patients in a collaborative process so that decision-making becomes one of a shared experience, and one in which the patient does not feel the burden upon themselves to make the decision alone. This move towards applying, or at least including, a more relational approach to decision-making, which focuses on emphasising virtues and moral character as key to ethical thinking, comes among the beginning of a resurgence in this area of thinking [46]. It is a shift away from the current rule-based deontological principles, such as the four pillars [47], which, despite widespread criticism [48–50], remain key to contemporary mainstream medical ethics. Our focus here has been on those virtues that emerged most prominently from our findings, but other virtuous notions may also be relevant here, for example, epistemic humility and/or patience to wait for a less emotionally-laden time to go over consent with patients.^3^

**Table 1:** Definitions of three virtuous approaches to be adopted during consent to clinical genetic testing

Virtue	Description of virtue	Practicing the virtue in the context of genetic testing	Illustrative Quote
Openness	The spirit of open communication; open-mindedness about decision-making and ethical views	Giving patients unrestricted access to the HCPs’ knowledge and information, even if that means HCPs telling patients they do not have all the answers; that they do not know all the information; or that the information is uncertain. Not hiding behind providing medical ‘certainties’ or informational answers to patients, but acknowledging and explaining the uncertain nature of genetic testing. Part of openness is also talking to patients about the way information might be shared - for research or to benefit relatives and considering this in light of patient’s relational (emotional, cultural etc) context.	‘Y*ou always tell your patient that’s what you’re going to do and you’re always transparent about what you are doing and why you’re doing it’*
Honesty	Refusing to fake the facts of reality	The HCP being sincere with patients, not overstating the potential of genetic testing or creating false expectations, and being upfront about the uncertainty which surrounds much genetic testing. This differs from information-provision in that HCPs make clear when they are uncertain ie., when there is no information to give per se, and also because *they have a conversation with patients*, rather than simply imparting knowledge	*‘I think if you’re honest about it then they don’t feel cheated’*
Trustworthiness	Being worthy of trust. People can count on you to do your best, to keep your word, and to follow through on your commitments	The HCP building a relationship with the patient such that the patient can rely and depend upon the HCP. In particular, the patient feels the HCP is treating them with respect, and that the HCP has considered the patient’s social, emotional and situational circumstances within their interactions with the patient	*‘I think it’s really important that your patient feels that they can trust the relationship that they’ve got with you’*

http://stthomassource.com/content/2017/04/09/virtueof-the-week-trustworthiness-3/

In spite of HCPs in genetic medicine recognising the value of adopting such process-led relational approaches to clinical genetic testing within their practices, such an approach to consent is not yet viewed as best practice in the field. As noted in our findings, one-off appointments for consent to genetic testing and long information sheets and consent forms are increasingly common in the NHS (and feature in the 100,000 genomes project). A move towards embedding virtuous principles in a more collaborative decision-making and thus more collaborative consent process also entails a move away from the perception that the ethical basis of consent is always trumped by the legal basis: more specifically, the perception that the *legally* protective way to acquire consent is to give patients as much information as possible. While we acknowledge that the Mongomery v Lanarkshire ruling might make it difficult for HCPs to feel secure in taking the approach we suggest in this paper, we have stressed that providing more information to patients does not necessarily mean better consent. We recommend that our approach is adopted in practice.

## Conclusions

Our approach provides a robust ethical framework suitable for HCPs conducting clinical genetic testing,^4^ More research is also needed that takes an explicitly virtue-ethics approach from the outset to move consent into the ‘right’ direction. We hope that this article, and others like it, can act as a concrete step towards inspiring discussion and raising awareness about alternative approaches to consent.

## Abbreviations

HCP: Healthcare professionals
IF: Incidental findings

## Footnotes

1 Broad consent provides only general information about the characteristics of genetic testing to individuals. It is commonly used in bio-banking, where it is impossible to foresee what research the genetic sample and information will be used for in the future. It is also used to tackle the problem that a test can produce IFs. This approach tiers, or ‘bins’, different types of IFs depending on factors such as clinical actionability, so that patients can chose which tier/bin they want to be told about.

2 We are aware that the interpretative nature of qualitative research means that others may have drawn out different HCP characteristics from our data set, meaning that any analysis will be limited to the interpretations of the authors. However, to enhance the confirmability of our interpretations, and to ensure rigour in our research methods, we analysed the data set within a team (including the duplicate coding by author’s 1 and 2, and then comparison of findings).

3 While other virtuous notions may be present, and seemingly prominent, in the data set provided here, openness, honesty and trustworthiness were by far the most prominent virtues emerging from the data set as a whole.

4 While concerns have been raised about the lack of clarity virtue ethics provides regarding how to adjudicate one virtue over another in practice, our intention is not to view these virtues as a set of rule base principles, but rather as a set of virtuous notions which HCPs can bring to their discussions with patients when seeking consent for clinical genetic testing. Any potential conflicts between virtues, if they indeed arise, would have to be considered in further research, informed by examples of real-life cases in which they emerge.
